# Study on the influence of reverse faulting on deformation of foundation pit retaining piles

**DOI:** 10.1038/s41598-023-44805-0

**Published:** 2023-10-14

**Authors:** Yungang Niu, Qiongyi Wang, Fenghai Ma

**Affiliations:** 1 College of Architecture and Engineering, Dalian University, Dalian, 116622 China; 2 School of Mechanics and Engineering, Liaoning Technical University, Fuxin, 123000 China

**Keywords:** Environmental sciences, Natural hazards, Materials science

## Abstract

The mechanical properties of soil in fault-fracture zone areas are diverse and complex. Deep excavation projects often encounter adverse geological conditions such as reverse faulting, which can lead to surface subsidence and collapses, posing significant challenges to excavation safety. Currently, there is limited research in the field of deep excavation engineering that analyzes the influence of reverse faulting on the deformation of retaining piles, and the existing research methods are not systematic enough. Therefore, this study aims to investigate the characteristics of how reverse faulting affects the deformation of retaining piles in deep excavation projects. Various research methods were employed, including numerical simulation, on-site monitoring, and orthogonal experiments, using a deep excavation project in Shenzhen as a case study. The results of the study indicate that reverse faulting exacerbates the deformation of retaining piles, causing the trend of increased deformation to shift upward. The upper part of the pile is significantly more affected than the lower part, and the overall deformation of the pile exhibits an approximate spoon-shaped curve distribution, with the maximum deformation occurring in the upper-middle section of the excavation. Under the influence of reverse faulting, the deformation of retaining piles is positively correlated with fault slip distance and fault dip angle, while it is negatively correlated with fault position. The growth rate of the maximum deformation of retaining piles, denoted as *r*(Δ*Z*_*max*_/Δ), increases approximately logarithmically with increasing fault slip distance and exponentially with increasing fault dip angle, but decreases approximately logarithmically with increasing distance from the fault to the excavation. An analysis of the sensitivity of fault slip distance, fault dip angle, and fault position to the maximum deformation of retaining piles was conducted. It was determined that the fault dip angle has the highest sensitivity, followed by fault slip distance, while fault position has the lowest sensitivity. Based on the fitting of 64 sets of orthogonal experimental data, a good linear relationship was established between the maximum deformation of retaining piles (*U*_*hm*_) and the indicator *η*($\theta \pi T/180^{^\circ } S$ ), leading to the development of a predictive model for the maximum deformation of retaining piles under the influence of reverse faulting. These research findings provide valuable insights and references for similar engineering projects.

## Introduction

As urban construction continues to develop, the utilization of underground space has attracted widespread attention. In urban engineering construction, the existence of special geological environments, such as active fault zones and fault slippage, has caused serious damage and threats to engineering^1–4^. The existence of fault slippage not only causes damage to engineering structures such as cross-fault buildings and underground engineering structures, but also poses a serious threat to the structural engineering in its affected area^5–9^. Excavation engineering located in the influence zone of a fault zone is easily affected by fault activity, leading to a series of adverse consequences such as displacement of the excavation support structure, uplift and cracking of the bottom plate, and damage to anti-seepage structural facilities. Therefore, fault activity poses a great threat to the safety of excavation engineering in its affected area. It is necessary to conduct research on the impact of fault activity on the deformation of excavation support piles to address this situation.

By analyzing the research of domestic and foreign scholars on foundation pit engineering and similar projects, this paper further consolidates the research foundation. Gao et al.^10^, in the context of a circular foundation pit excavation support project in the sandy soil layer of the West Anchorage of Humen Second Bridge, proposed a novel method for parameter calibration based on continuous wall displacement monitoring data. They also developed modeling and computational theories for the excavation support process of the circular foundation pit. This allowed them to obtain insights into the stress-deformation mechanisms of the soil layers and the supporting structures during the excavation and support of the sandy soil layer in the circular foundation pit.Zhang et al.^11^ proposed a calculation method for the deformation of retaining walls considering the spatiotemporal effects. The method was based on theoretical analysis, numerical calculations, and field measurements. The researchers used the influence coefficient of pit corner effect and the equivalent horizontal resistance coefficient to measure the impact of spatiotemporal effects on support deformation. Qin et al.^12^ mainly studied the influence of the reinforced area within the deep silt layer pit, the embedding depth of support piles, and the stiffness of support piles on the deformation law of foundation pits, and proposed corresponding deformation control measures. Mei et al.^13^, through statistical analysis of field measurement data from the excavation of ten subway stations in Xi'an, investigated the spatiotemporal characteristics of surface settlement and pile lateral deformation caused by deep excavation in expansive loess. Furthermore, they delved into the deformation mechanisms of excavations in loess regions, which is of significant importance for enhancing the design theory of excavations in this area. Zhang et al.^14^, in response to the safety requirements for deep excavation construction imposed by the construction of large-span high-speed railway arch bridges, based on the Xu-Honghe River Special Bridge Project of the Xuyan High-speed Railway, employed numerical simulation methods and combined them with on-site measurement data. They conducted a systematic study on the deformation and stress characteristics of steel sheet piles during the construction of deep excavation support structures for seismic fault zones in large-span railway arch bridge projects. Lei et al.^15^ conducted a numerical simulation of fault slip instability under the influence of horizontal structural stress using the 21,221 working face in the Yima Coalfield as the engineering background. They studied the evolution characteristics of the displacement field of a fault structure with a dip angle of 75° under mining disturbance.Baziar et al.^16^ studied the interaction between shallow foundations and reinforced soil under normal and reverse faults, and verified the accuracy of numerical simulation results through experiments conducted at the University of Dundee and Waseda University. Liu et al.^17–19^ studied the deformation of the strata, tunnel strain, and failure characteristics caused by a 75° reverse fault through model tests. Based on this, the force and deformation mechanism of a tunnel under the influence of sliding along a 75° dipping normal fault was studied through indoor model tests. Subsequently, the finite element method was used to compare and study the influence of different lining section shapes on the structural force and deformation of the tunnel under fault sliding. Triantafyllaki et al.^20^ achieved important research results in numerical analysis of the response of deep-sea buried pipeline structures crossing active normal and reverse faults. Jin et al.^21^, based on a deep excavation project in a karst and fractured zone formation near a subway station in Nanjing, comprehensively analyzed issues related to insufficient embedment depth of the ground-anchored wall, which led to sudden water inflow at the pit bottom and pit deformation. Subsequently, they conducted optimization design of the protective structure and implemented measures for controlling the water inflow. Post-assessment results demonstrated significant improvements resulting from these measures.Liu et al.^22^, through the statistical analysis of 42 engineering cases of foundation pit projects, investigated the influence of significant factors on the vertical and horizontal displacements of tunnels. They proposed a comprehensive prediction index for the horizontal displacement of tunnels and presented empirical prediction formulas for tunnel horizontal displacement under three different geological conditions. Xue^23^ based his research on a deep foundation pit project at a station on Changchun Metro Line 5. Using time series, he obtained training data and integrated the GA algorithm into the PSO algorithm for parameter optimization. By combining grey theory, he established a deformation time series prediction model for the foundation pits based on Grey Least Squares Support Vector Machine (Grey-LSSVM).

In summary, through the analysis of research findings from multiple scholars in the field of foundation pit engineering and fault activities, it can be observed that there is currently limited research in the field of foundation pit engineering on the impact of faults on the deformation of retaining piles, and even less research analyzing the influence of reverse faulting on the deformation of retaining piles. Therefore, conducting research in this area holds significant importance for both theoretical understanding and practical engineering applications. This study focuses on a specific foundation pit project in Shenzhen City. By comparing and analyzing numerical simulations and field measurements of the foundation pit retaining piles, the reliability of finite element calculations is validated. The study specifically emphasizes the investigation of the deformation effects of the foundation pit retaining piles in the influence zone of the footwall affected by reverse faults. The main research content includes analyzing the deformation characteristics of the retaining piles under the influence of reverse fault, considering different fault slip amounts, dip angles, and positions, and assessing the sensitivity of these parameters to the maximum deformation of the retaining piles. Finally, through fitting 64 sets of orthogonal experimental data, a prediction model and a project-specific prediction formula for the maximum deformation of the foundation pit retaining piles in the influence zone of the footwall affected by reverse fault are determined.

## Engineering overview

### Project design overview

**Figure 1:**
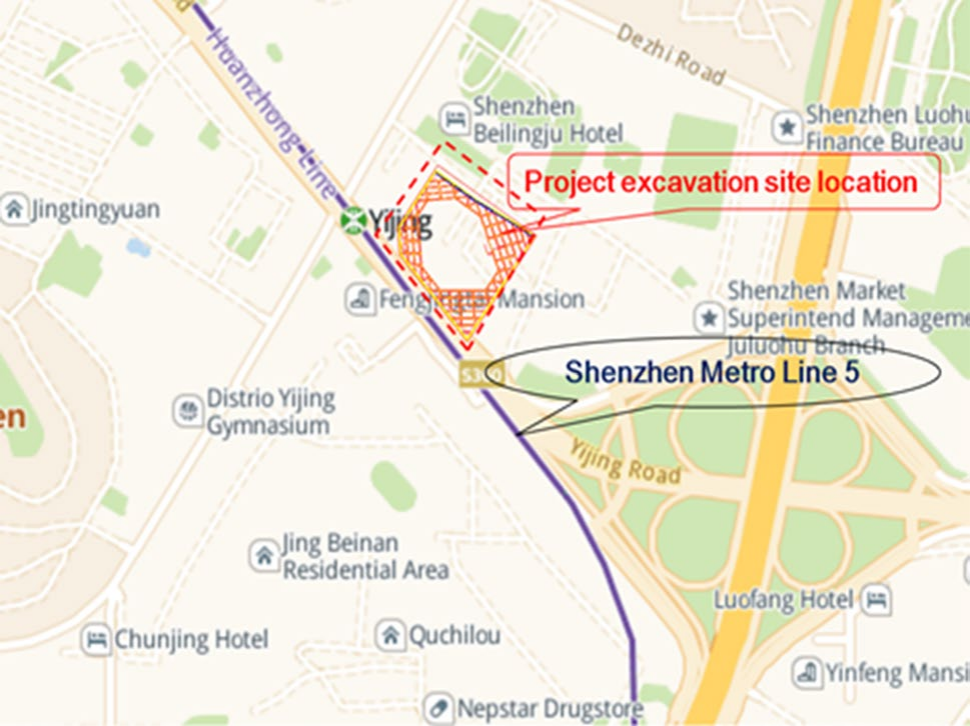
Foundation pit excavation plane location.

**Figure 2:**
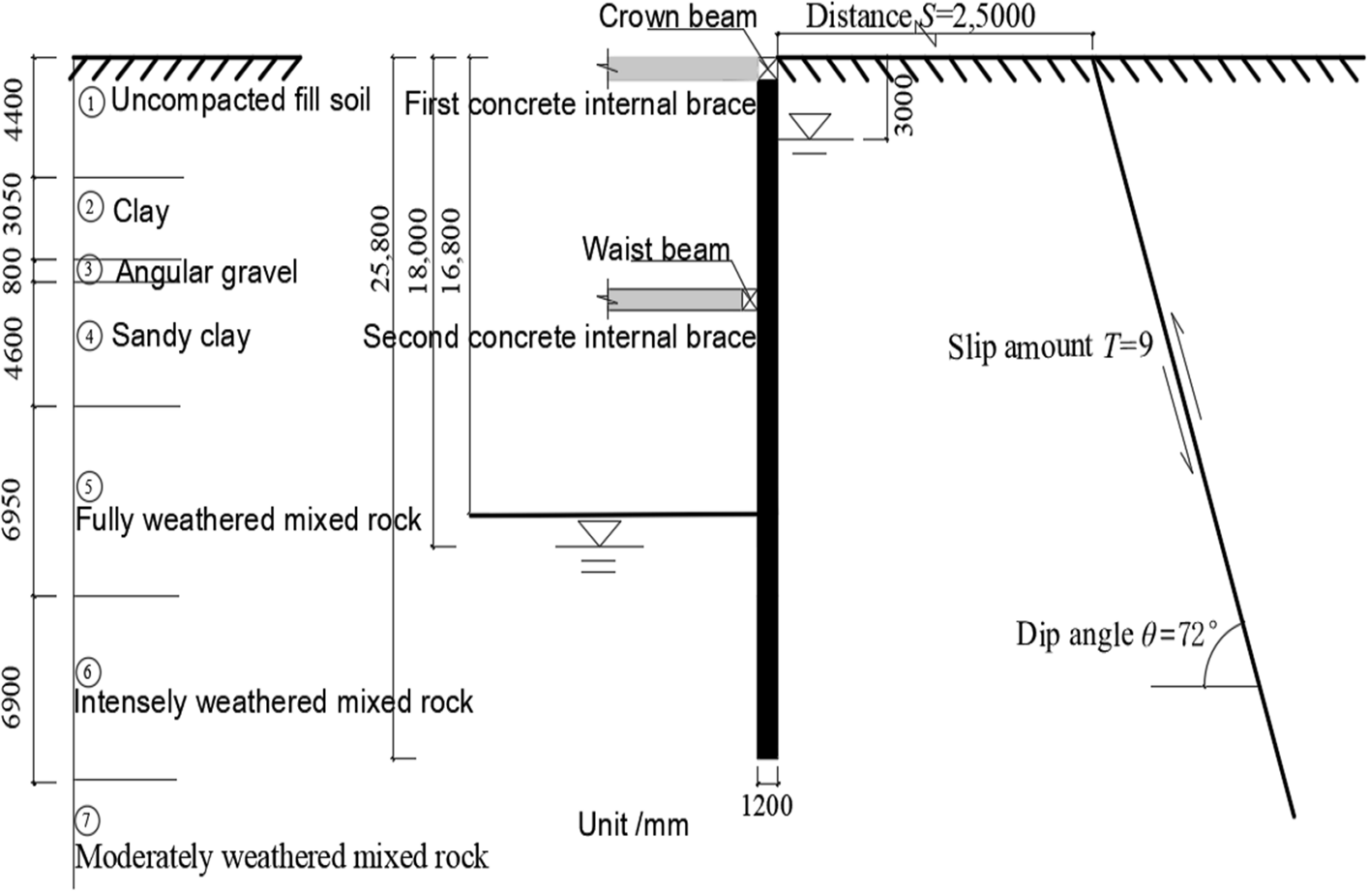
The schematic cross-section of the foundation pit and the reverse fault.

This project is located on the southeast side of Yijing Station, Line 5 of the Shenzhen Metro, in the Luohu District of Shenzhen. The plan view of the foundation pit is shown in Fig. 1^24^. The safety level of the excavation support system is classified as Level 1. The total area of the foundation pit is approximately 8900 m^2^, with a perimeter of about 380 m. The maximum excavation depth is 16.8 m. The support structure consists of piles and internal bracing, with two rows of internal bracing at a spacing of 8.5 m. The diameter of the retaining piles and column piles is both 1.2 m, with a length of 25.8 m for the retaining piles and 20.0 m for the column piles. The cross-sectional dimensions of the crown beam, waist beam, and support beams are all 1.0 m × 1.2 m. The concrete strength grade for the retaining piles, support beams, connecting beams, and column piles is C30. The cross-sectional dimensions and physical–mechanical parameters of the support structure are shown in Table 1. The schematic cross-section of the foundation pit and the reverse fault is shown in Fig. 2.

**Table 1:** The cross-sectional dimensions and physical–mechanical properties of the support structure.

Name	Material	Section dimensions (mm)	Unit weight (kg/m^3^)	Elastic modulus (GPa)	Internal friction angle (◦)	Poisson's ratio
Retaining pile (diaphragm wall)	C30	Thickness 880	2400	30	26	0.2
Column pile	C30	Ø1200	2400	30	26	0.2
Crown beam	C30	1000 × 1200	2400	30	26	0.2
Support beam	C30	1000 × 1200	2400	30	26	0.2
Waist beam	C30	1000 × 1200	2400	30	26	0.2

### Site geological condition

The site is located within the South China Series Bijiashan Formation (NhB) and is influenced by the F1322 Fault Zone (Shehe Cang-Henggang-Luohu Fault Zone). The fault zone is the main fault within the fault system, with a length of 38 km and a width ranging from 7 to 70 m, reaching a maximum width of approximately 267 m. It strikes in the range of NW 25°–55°, dips towards the southeast at an angle of 60°–80°, and is a high-angle reverse fault active during the Holocene. Due to tectonic movements at different periods, the fill materials in the fault zone include clay minerals, talc, quartz, etc., with relatively poor mechanical properties, and the fault surfaces are rough.

The soil parameters selected based on the investigation report are listed in Table 2^24^. The simplified stratigraphic structure and thickness of the site from top to bottom are as follows: (1) Uncompacted fill soil with a thickness of 4.4 m; (2) Clay with a thickness of 3.05 m; (3) Angular gravel with a thickness of 0.8 m; (4) Sandy clay with a thickness of 4.6 m; (5) Completely weathered rock with a thickness of 6.95 m; (6) Strongly weathered mixed rocks with a thickness of 6.90 m; (7) Moderately weathered mixed rocks, which have not been penetrated. Affected by the F1322 Fault, the bedrock at the site is relatively fragmented, and local occurrences of green clay alteration can be observed. The stability of the area where the site is located is relatively good, belonging to a stable region.

**Table 2:** Calculation parameters of soil layer.

Stratigraphic (genetic)	Natural weight (kN/m^3^)	Tri-axial test secant modulus (MPa)	Secant modulus of elasticity (MPa)	Unloading modulus of elasticity (MPa)	Poisson's ratio	Cohesion (kPa)	Internal friction angle (◦)	Permeability coefficient (m/day)
① Uncompacted fill soil	19.0	2	2	6	0.35	14	15	5
② Clay	18.5	2.8	2.8	8.4	0.33	25	18	0.1
③ Angular gravel	19.5	6	6	18	0.28	0	36	25
④ Sandy clay	21.0	8	8	24	0.26	25	22	5
⑤ Fully weathered mixed rock	20.0	16	16	48	0.24	23	28	0.1
⑥ Intensely weathered mixed rock	20.5	25	25	75	0.23	20	32	0.5
⑦ Moderately weathered mixed rock	21.0	70	70	210	0.22	200	50	0.5

## Three-dimensional numerical simulation analysis

### Numerical calculation model

**Figure 3:**
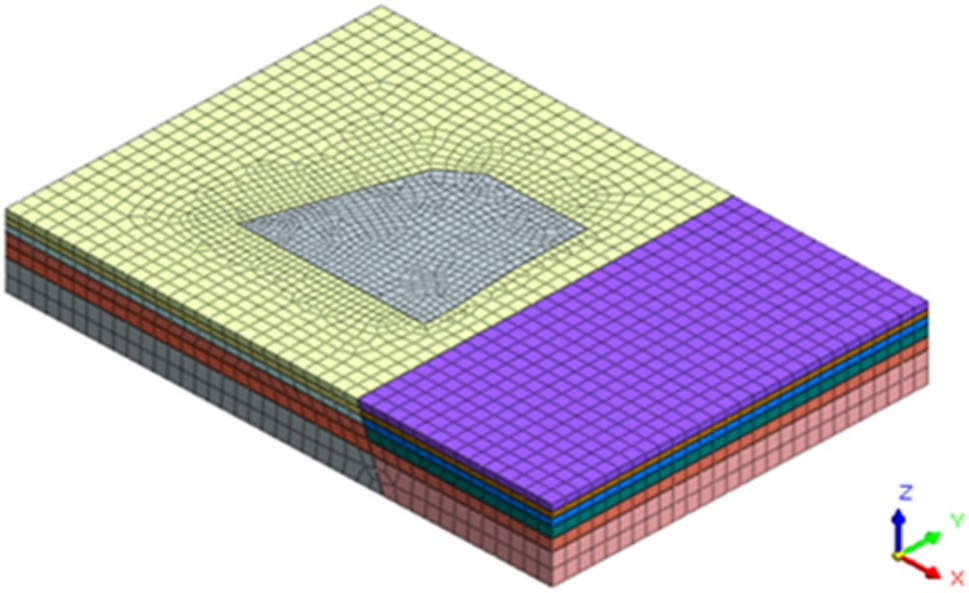
Three-dimensional numerical calculation model.

The MIDAS GTS NX finite element analysis software was used to establish a three-dimensional numerical calculation model. In order to reduce boundary effects and fully reflect the fault slip phenomenon, the numerical calculation model size was selected as length × width × height = 320 m × 220 m × 45 m, as shown in Fig. 3. Assuming that each soil layer follows an ideal elastoplastic model and obeys the Mohr–Coulomb strength criterion, the support structure of the foundation pit adopts a linear elastic model. The retaining piles of the foundation pit are simulated using an equivalent earth retaining wall model, with an equivalent thickness of 0.88 m. The strata were simulated using three-dimensional solid elements, while the retaining structure was simulated using two-dimensional plate elements. The column piles, crown beams, waist beams, and support beams were simulated using one-dimensional beam elements.

**Figure 4:**
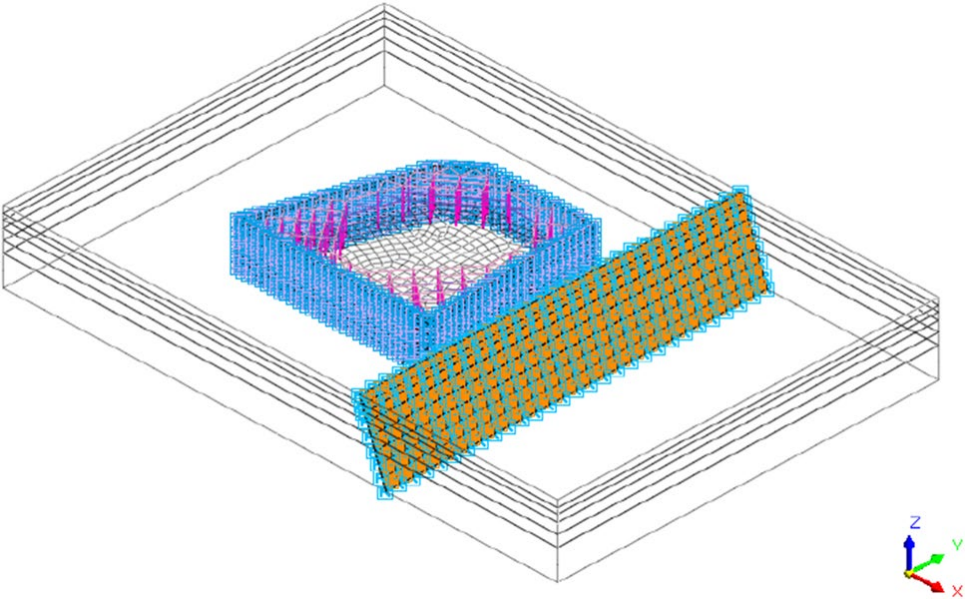
Foundation pit supporting system and interface elements.

In order to accurately depict the water cutoff effect of the interlocking piles and cutoff curtain, the MIDAS GTS NX software was used to simulate the cutoff curtain using interface contact elements, with a permeability coefficient set to 0. For computational simplicity, the fault plane was assumed to have a regular geometric shape and was also simulated using the interface contact elements in the MIDAS GTS NX software. The interface element parameters were automatically calculated by the finite element interface assistant based on the parameters of the rock and soil on both sides of the contact plane, and the comprehensive judgment resulted in the interface calculation parameters shown in Table 3. The excavation support system and interface element settings are shown in Fig. 4.

**Table 3:** Contact surface calculation parameters.

Slip plane	Normal stiffness (kN/m^3^)	Shear stiffness (kN/m^3^)	Internal friction angle (°)	Cohesion (kPa)
Soil–soil interface element	5.6 × 10^4^	5.6 × 10^3^	10	13
Rock–rock interface element	3.2 × 10^6^	3.2 × 10^5^	16	22

### Boundary conditions and analysis scenarios

**Figure 5:**
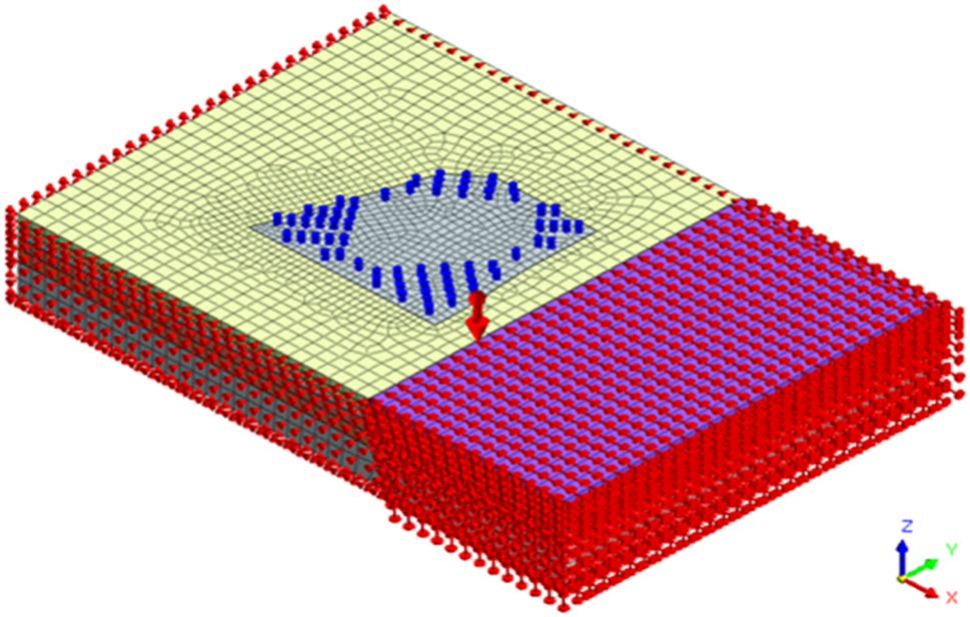
Boundary conditions and loading conditions.

Before conducting finite element dynamic simulation calculations, horizontal constraints were applied around the model, and vertical constraints were applied at the bottom. By fixing the free movement of the lower part of the fault, a certain forced displacement is applied to the upper part of the fault to analyze the deformation effects of the foundation pit retaining piles in the influence zone of the footwall affected by the reverse fault. Similarly, by applying appropriate constraints to the corresponding boundaries, the deformation effects of different fault dip angles and fault positions on the retaining piles were simulated under certain slip amount. The initial fault parameters were set as follows: fault distance from the excavation is 25 m, fault dip angle is 72°, and fault amount is 9 mm. The boundary constraints and loading conditions for this simulation are shown in Fig. 5.

### Analysis of numerical simulation results

**Figure 6:**
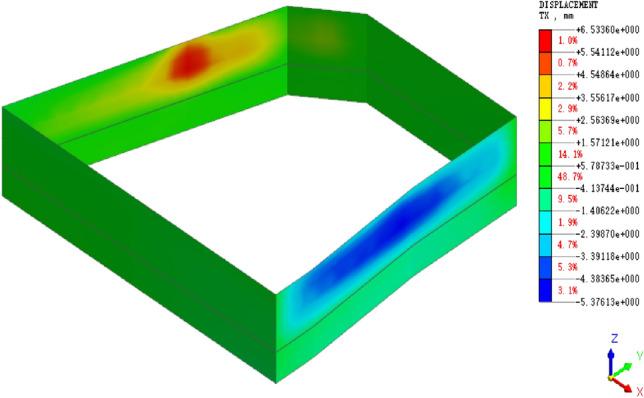
Cloud map of horizontal displacement of retaining piles after excavation completion.

**Figure 7:**
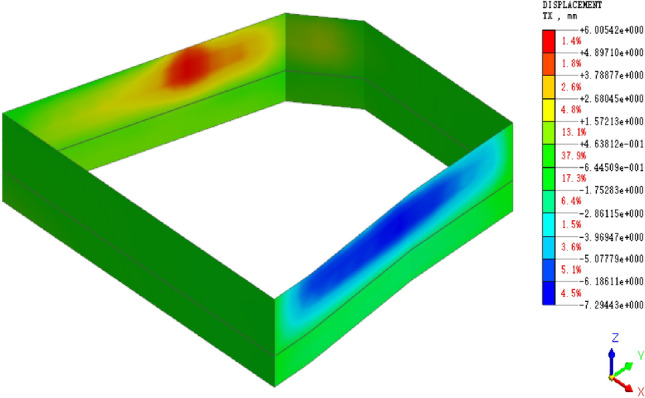
Cloud map of horizontal displacement of retaining piles under bonded sliding of the reverse fault.

Through finite element calculation, the horizontal displacement cloud maps of the retaining piles under completion of excavation and bonding slip along the reverse fault are obtained, as shown in Figs. 6 and 7.

**Figure 8:**
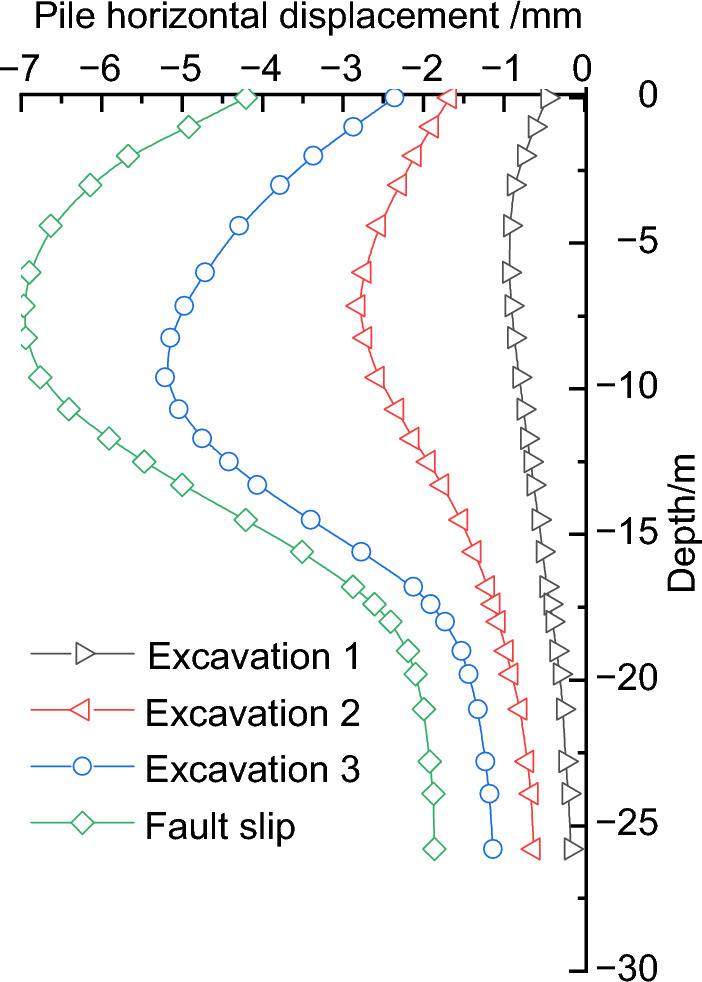
Analysis of numerical calculation results of retaining wall deformation.

Extracting the calculation results of the horizontal displacement of a point (JCX5) on the side of the retaining pile near the fault, we obtain the deformation curve of the retaining pile, as shown in Fig. 8. It is observed that the deformation of the retaining pile exhibits a distribution that gradually expands inward from top to bottom, with the maximum deformation occurring near the excavation face. As the pit excavation progresses, the deformation of the retaining pile continuously increases, and the rate of maximum deformation growth also increases. Simultaneously, the position of the maximum deformation gradually shifts downward. When the pit excavation is completed, the maximum horizontal displacement of the retaining pile is 5.21 mm, with the maximum deformation occurring in the middle-lower part of the pit.

When some bonding slip occurs along the reverse fault, the retaining pile undergoes further deformation due to the compressive action between the fault surfaces. The trend of increased deformation shifts towards the upper part, and the effect of deformation is more significant in the upper part of the pile. The maximum deformation occurs near the middle-upper part of the pit, and the overall deformation of the retaining pile exhibits an approximate spoon-shaped distribution. At this stage, the maximum horizontal displacement of the retaining pile is 7.15 mm.

### Comparison and analysis of numerical and field monitoring results

**Figure 9:**
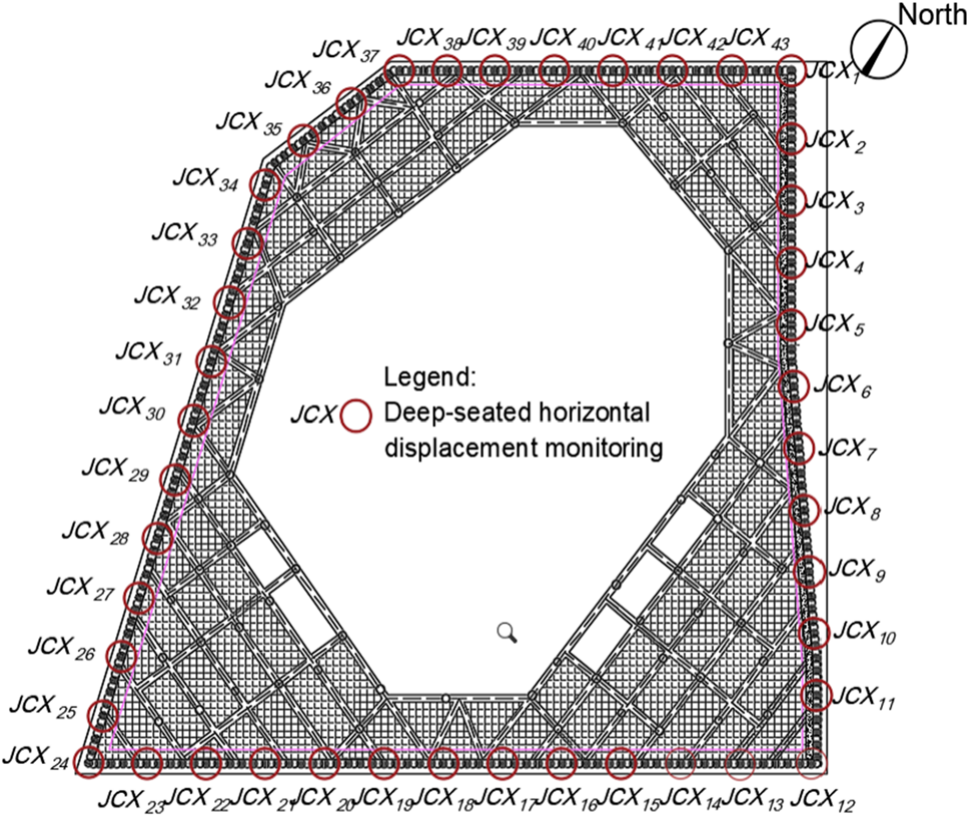
Plane layout of measured points.

The on-site measurements for this project include the deep-seated horizontal displacement of the retaining piles. Monitoring points were arranged with one monitoring point every five interlocking piles, totaling approximately 43 monitoring points, as shown in Fig. 9. The on-site monitoring utilized advanced automated equipment provided by Southern Intelligent Precision Measurement. The monitoring frequency was determined in accordance with the "Shenzhen Engineering Construction Standard—Foundation Pit Support Technical Standard (SJG 05-2020)". The lateral displacement of the station was monitored in three stages during the excavation phase: the first stage had a monitoring frequency of every two days, the second stage was monitored daily, and the third stage increased to twice daily. After the completion of pouring the pit bottom, the monitoring frequency for lateral displacement was adjusted to every 3 days.

**Figure 10:**
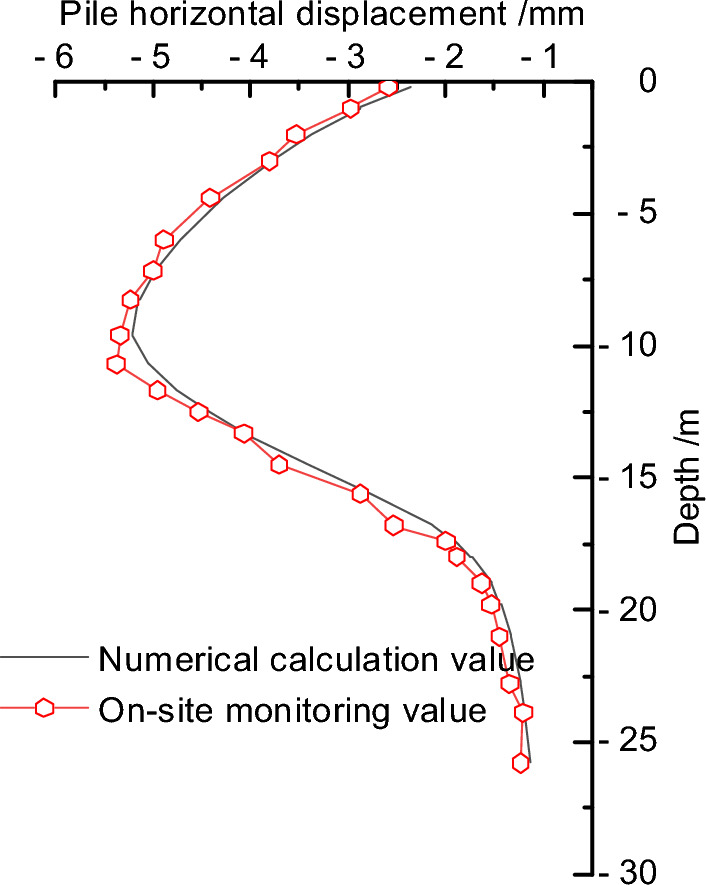
Comparison of deformation monitoring of retaining pile.

The measured results of the horizontal displacement of the pile body at the monitoring point near the fault side of the excavation pit (*JCX5*) were extracted, and a comparative analysis was conducted with the numerical calculation results, as shown in Fig. 10. It should be noted that the measured results may be influenced by factors such as unconsidered stratified excavation, surrounding surcharge, and delayed reinforcement of the support system, which may lead to an enlargement of the deformation to some extent and hence overestimate the results. Overall, the numerical results from both methods exhibit consistent patterns, indicating that finite element analysis can to a certain extent reflect the actual situation. Therefore, this study will focus on conducting deformation analysis of the retaining pile under the influence of the reverse fault using the finite element analysis approach.

## Analysis of the effects of fault parameters

In order to study the influence of fault parameters on the deformation of retaining piles, this section uses the method of controlling variables, assuming that the other parameters remain unchanged, and analyzes the impact of one parameter variation. Through finite element analysis, the effects of three parameters—fault slip, fault dip angle, and fault position—on the deformation of retaining piles are studied. The initial fault parameters are set as follows: fault slip amount is 9 mm, fault dip angle is 72°, and fault distance from the excavation is 25 m.

### Analysis of the effect of fault slip amounts

**Figure 11:**
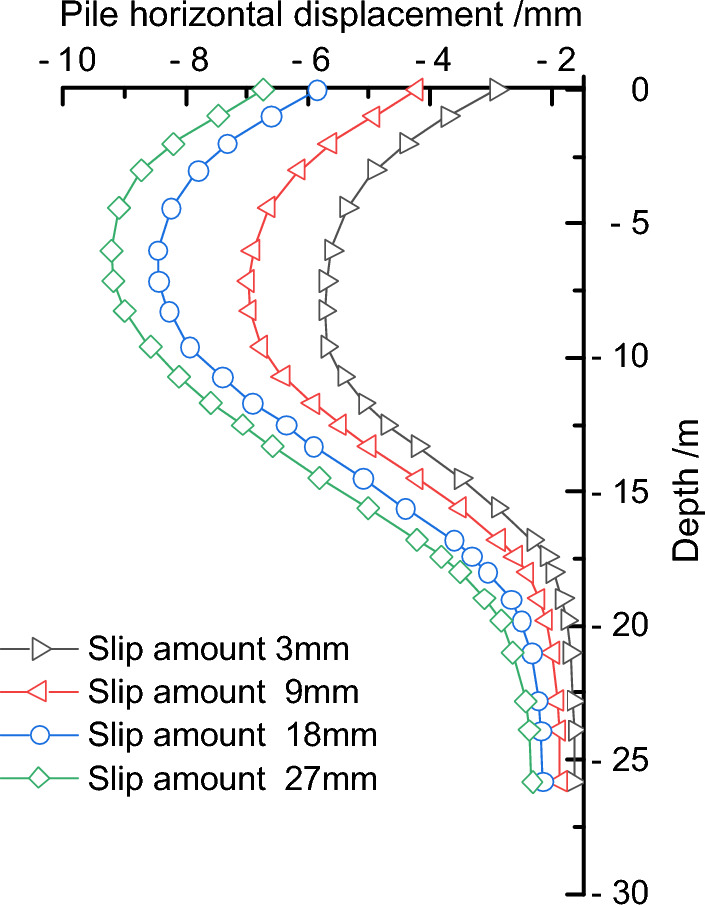
Comparison of retaining pile deformation under different slip amounts.

Keeping the fault dip angle and position unchanged, the influence of fault slip amount on the deformation of retaining piles was studied by sequentially adjusting the fault slip amount to 3 mm, 9 mm, 18 mm, and 27 mm. By finite element calculation, the deformation law curves of the retaining piles under different slip amounts were obtained, as shown in Fig. 11. Under different slip amounts, the deformation of the retaining piles basically followed the spoon-shaped curve law. As the slip amount increases, the influence of the fault's compressive force on the retaining piles intensifies, resulting in an increasing trend of deformation. In a certain range, the maximum deformation growth rate first increased and then decreased. In addition, the position of the maximum deformation of the retaining pile also continuously moved upward, and the deformation effect of the upper part of the pile body was significantly higher than that of the lower part.

**Figure 12:**
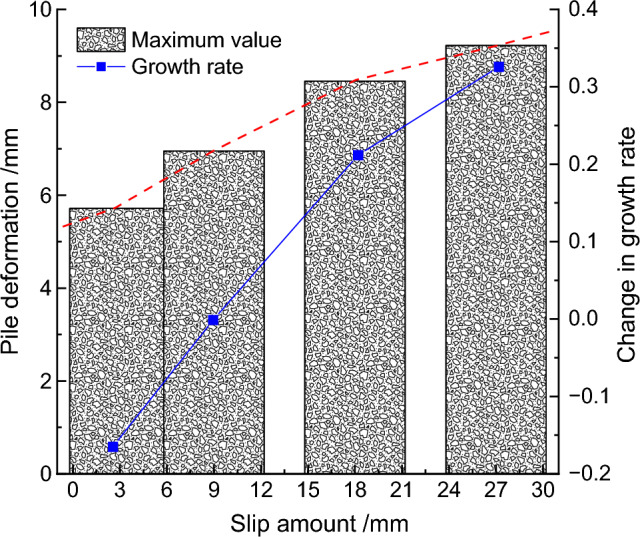
Analysis of maximum deformation variation of retaining pile under different slip amounts.

To further quantify the results, the maximum deformation values of the retaining pile under different slip amounts were extracted, and the curve of the maximum deformation of the retaining pile was obtained, as shown in Fig. 12. With the increase of fault slip amount, the maximum deformation of the retaining pile increased non-linearly. Meanwhile, taking the slip amount of 9 mm as the reference, introducing the maximum deformation growth rate *r*_*1*_ (Δ*Z*_*max*_/Δ), it was found that the maximum deformation growth rate of the retaining pile increased approximately logarithmically with the increase of fault slip amount.

### Analysis of the effect of fault dip angles

**Figure13:**
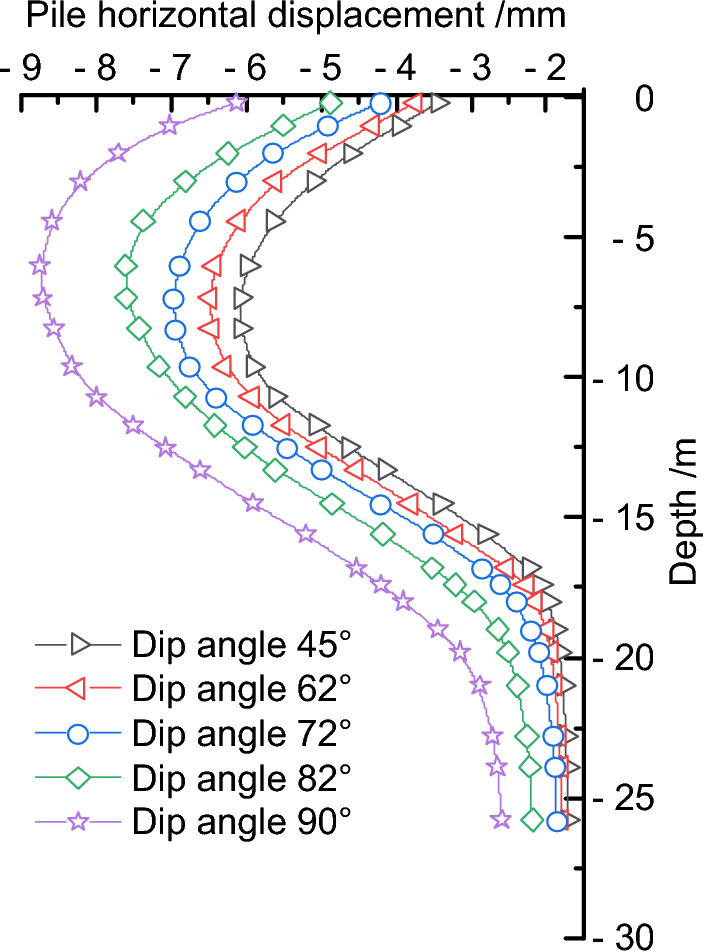
Comparison of retaining pile deformation under different dip angles.

Keeping the fault slip amount and position unchanged, the fault dip angle was adjusted in sequence to 45°, 62°, 72°, 82°, and 90° to study the deformation characteristics of the retaining piles with the increase of fault dip angle. The deformation impact law curves of the retaining piles under different fault dip angles were obtained through finite element calculations, as shown in Fig. 13. Under different fault dip angles, the deformation curve of the retaining pile basically conforms to the spoon-shaped curve law. With the increase of fault dip angle, the influence of the geological pressure on the retaining pile becomes stronger, leading to an increasing trend of the pile deformation and the growth rate of the maximum deformation. In addition, the maximum deformation position of the retaining pile also continuously shifts upward, and the deformation impact on the upper part of the pile is more significant than that on the lower part. Especially at large dip angles, the deformation of the retaining pile increases significantly, which poses a great threat to the excavation engineering.

**Figure 14:**
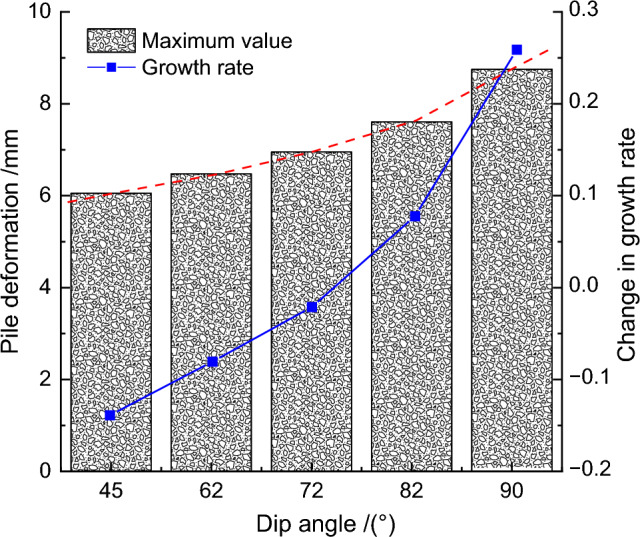
Analysis of maximum deformation variation of retaining pile under different dip angles.

To further quantify the results, the maximum deformation values of the retaining pile under different fault dip angles were extracted, and the maximum deformation impact law curve of the retaining pile was obtained, as shown in Fig. 14. With the increase of fault dip angle, the maximum deformation of the retaining pile increases nonlinearly. Meanwhile, taking the fault dip angle of 72° as the baseline, the maximum deformation growth rate *r*_*2*_ of the retaining pile was introduced, and it was found that the maximum deformation growth rate of the retaining pile approximately follows an exponential function with the increase of fault dip angle. Therefore, even with a small fault slip in a high dip angle reverse fault, it still has a significant impact on the deformation of the retaining pile.

### Analysis of the effect of fault locations

**Figure 15:**
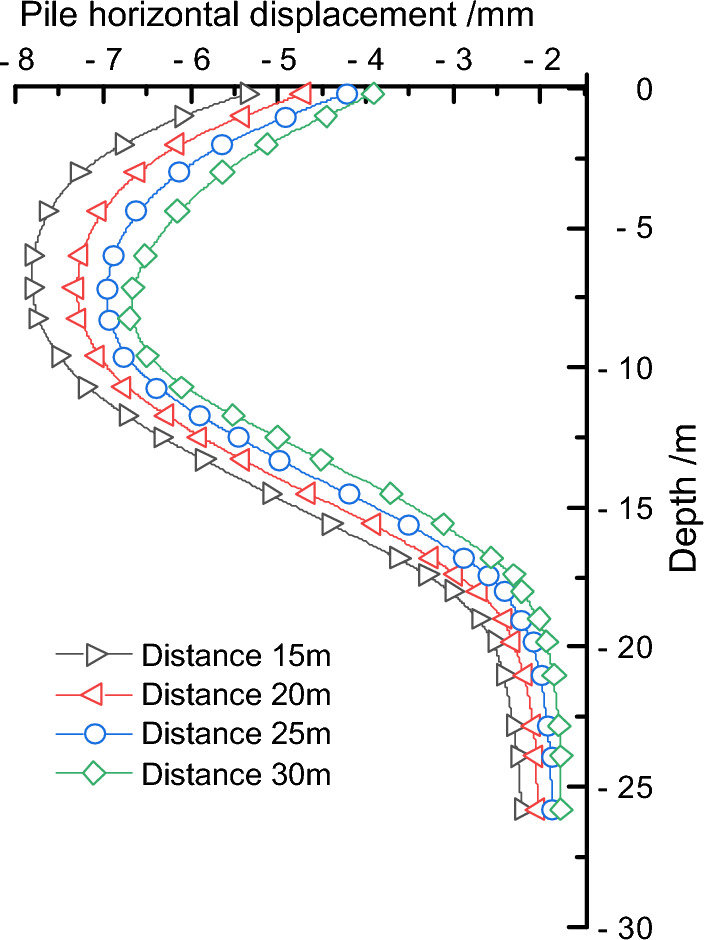
Comparison of retaining pile deformation at different locations.

Keeping the fault slip amount and dip angle unchanged, the distance between the fault and the foundation pit was sequentially adjusted to 15 m, 20 m, 25 m, and 30 m to study the deformation characteristics of the retaining piles as the fault distance from the pit increased. Through finite element calculation, the deformation influence curves of the retaining piles under different fault positions were obtained, as shown in Fig. 15. It was found that under different fault positions, the deformation of the retaining pile basically followed the spoon-shaped curve rule. With the increase of the distance between the fault and the pit, the influence of the stratum extrusion on the retaining pile weakened, resulting in a decreasing trend in deformation and a gradual decrease in the maximum deformation reduction rate. In addition, the maximum deformation position of the retaining pile also continuously moved downward, and the deformation influence on the upper part of the pile body remained significantly higher than that on the lower part.

**Figure 16:**
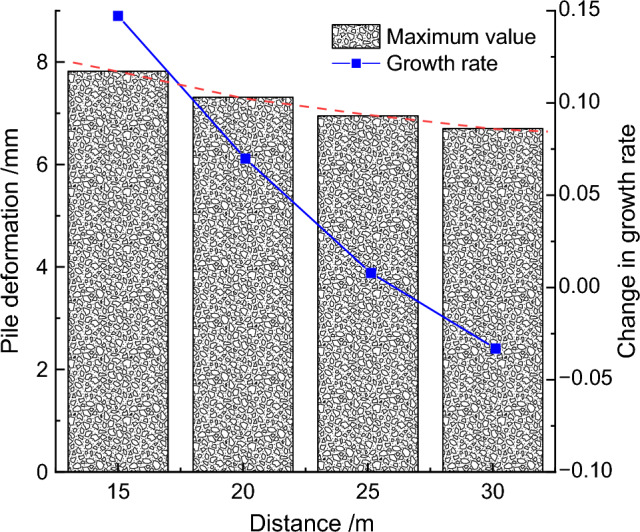
Analysis of maximum deformation variation of retaining pile under different locations.

To further quantify the impact, the maximum deformation values of the retaining pile were extracted at different fault positions, and the maximum deformation influence rule curve of the retaining pile was obtained, as shown in Fig. 16. It was found that as the distance between the fault and the pit increased, the maximum deformation of the retaining pile decreased nonlinearly. At the same time, taking the fault distance of 25 m from the pit as the reference, the maximum deformation growth rate *r*_*3*_ of the retaining pile was introduced. It was found that the maximum deformation growth rate of the retaining pile decreased approximately logarithmically with the increase of the fault distance from the pit, and when the fault distance from the pit reached a certain distance, the impact of this fault was very small.

## Sensitivity analysis

### Theoretical analysis

Assuming a set of variables, with the dependent variable *Y* being determined by *n* independent variables *X*_*1*_, *X*_*2*_, … , *X*_*n*_. The function relationship between *Y* and *X* can be expressed as: *Y* = *f*(*X*_*1*_, *X*_*2*_, … , *X*_*n*_). Under a certain condition, the independent variables are set as reference variables *X'*, resulting in a reference dependent variable *Y'* = *f*(*X'*_*1*_, *X'*_*2*_, …, *X'*_*n*_). The analysis involves examining the deviation degree and trend of the dependent variable relative to the reference dependent variable, known as sensitivity analysis^25^.

In order to compare the sensitivity of multiple independent variables, non-dimensional sensitivity functions and sensitivity factors are defined. The sensitivity function *P*_*i*_ (*X*_*i*_) is defined as:

1 $$P_{i} (X_{i} ){ = }\left| {\frac{{df(X_{i} )}}{{d(X_{i} )}}} \right|\frac{{X_{i} }}{Y}(i = 1,2,3...)$$

By substituting the baseline independent variable *X'*_*i*_ into Eq. (1), the sensitivity factor *P*_*i*_ of the independent variable *X*_*i*_ can be obtained. The larger the sensitivity factor, the stronger the sensitivity of the dependent variable to the independent variable *Xi* under the baseline state; conversely, the weaker the sensitivity.

### Theoretical results analysis

To clarify the sensitivity of the maximum deformation of the foundation pit retaining pile to the three parameters of fault slip amount, fault dip angle, and fault position, this section conducts a sensitivity analysis on the maximum deformation value of the foundation pit retaining pile with fault slip amount, fault dip angle, and fault position as independent variables and the maximum deformation of the retaining pile as the dependent variable.

#### Sensitivity analysis of fault slip amount *T*

**Figure 17:**
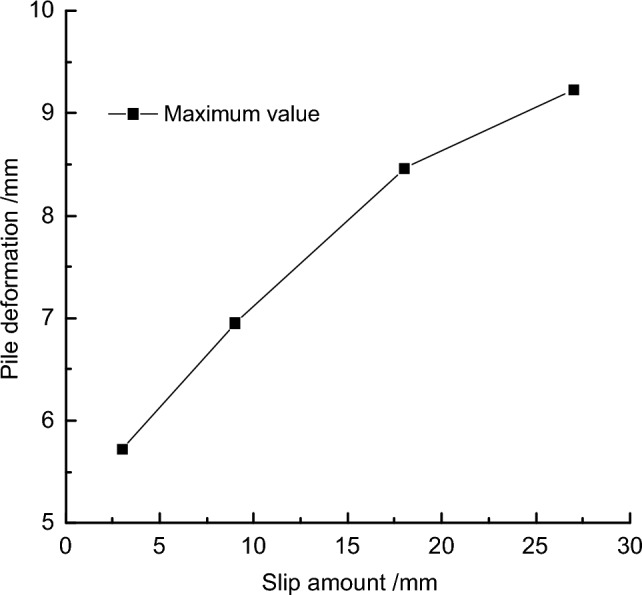
Relationship curve between pile maximum deformation and slip amount.

Keeping the fault dip angle and fault location constant, the maximum deformation values of foundation pit retaining pile were extracted for different fault slip amount values, and the relationship between the maximum deformation of retaining pile and fault slip amount is shown in Fig. 17. The maximum deformation of retaining pile increases with the increase of fault slip amount. A quadratic function is fitted to the relationship curve between the maximum deformation of retaining pile and fault slip amount, which is:

2 $$y = { - }0.0038T^{2} + \, 0.2617T + \, 4.9487$$

From Eq. (2), the sensitivity function can be obtained as:

3 $$P(T) = \left| { - 0.0076T + 0.2617} \right| \times \frac{T}{y}$$

Substituting the baseline value *T* = 9 mm into Eq. (3), the sensitivity factor of the maximum deformation of the retaining pile is *P*_*T*_ = 0.251.

#### Sensitivity analysis of fault dip angle *θ*

**Figure 18:**
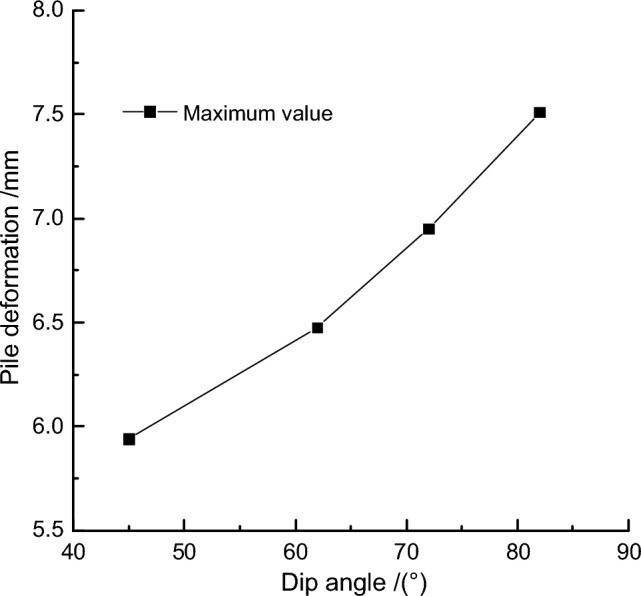
Relationship curve between pile maximum deformation and dip angle.

Controlling the fault slip amount and fault position, the maximum deformation value of the retaining pile in the foundation pit is extracted at different fault dip angles, and the relationship curve between the maximum deformation of the retaining pile and the fault dip angle is shown in Fig. 18. The maximum deformation of the retaining pile increases with the increase of the dip angle, and the quadratic function fitting curve of the relationship between the maximum deformation of the retaining pile and the dip angle is:

4 $$y = \, 0.0004\theta^{2} - \, 0.013\theta + \, 5.6583$$

where *θ* is calculated based on its value without considering its unit. The sensitivity function can be obtained from formula (4):

5 $$P(\theta ) = \left| {0.0008\theta - 0.013} \right| \times \frac{\theta }{y}$$

Substituting the baseline value *θ* = 72° into formula (5), the sensitivity factor of the maximum deformation of the retaining pile is obtained as *P*_*θ*_ = 0.462.

#### Sensitivity analysis of fault location *S*

**Figure 19:**
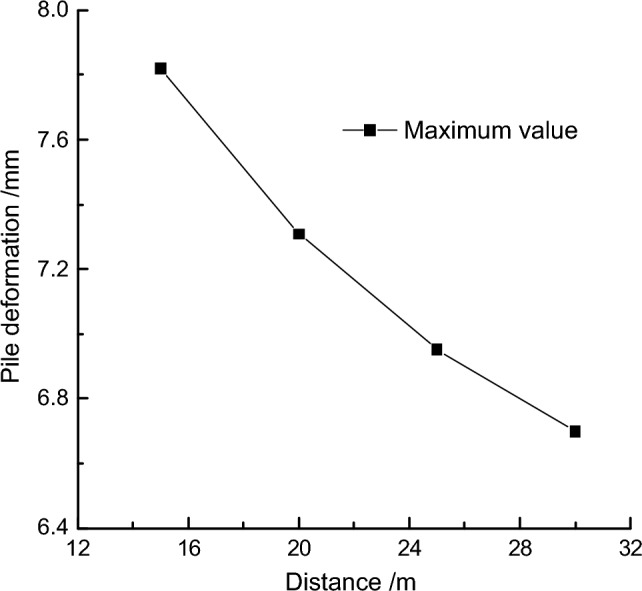
Relationship curve between pile maximum deformation and distance.

Controlling the fault slip amount and fault dip angle, different maximum deformation values of the retaining pile were extracted for different fault locations. The relationship between the maximum deformation of the retaining pile and the fault location is shown in Fig. 19. As the distance between the fault and the foundation pit increases, the maximum deformation of the retaining pile decreases. The quadratic function fitting the relationship between the maximum deformation of the retaining pile and the fault location is:

6 $$y = \, 0.0026S^{2} - \, 0.1903S + \, 10.093$$

The sensitivity function can be obtained from formula (6):

7 $$P(S) = \left| {0.0052S - 0.1903} \right| \times \frac{S}{y}$$

Substituting the base value *S* = 25 m into formula (7), the sensitivity factor of the maximum deformation of the retaining pile can be obtained as: *P*_*S*_ = 0.217.

In summary, under the influence of a reverse fault, the sensitivity factors of the maximum deformation of the retaining piles to various fault parameters are shown in Table 4. Among them, the fault dip angle has the highest sensitivity factor on the maximum deformation of the retaining piles, followed by the fault slip amount, while the influence of fault position is the smallest. All three parameters have a significant impact on the maximum deformation of the retaining piles. Therefore, in the practical assessment of the impact of reverse faulting on the deformation of excavation support piles, it is necessary to comprehensively consider the influence of various factors related to the fault.

**Table 4:** List of sensitive factors.

Indicators	Maximum deformation sensitivity factor of retaining piles *P*
Slip amount *T*	0.251
Dip angle *θ*	0.462
Position *S*	0.217

## Maximum deformation prediction model for retaining piles

In this section, an orthogonal experimental method^26–28^ was used to analyze a numerical simulation experiment with three factors, including fault slip amount, fault dip angle, and fault position, with four levels each, as shown in Table 5. Considering that the influence of a reverse fault on the maximum deformation of the retaining piles in the influence zone of the footwall is positively correlated with the fault slip amount and fault dip angle, while negatively correlated with the distance between the fault and the foundation pit, *η* ($\theta \pi T/180^{^\circ } S$) was chosen as the predictive indicator for the maximum deformation *U*_*hm*_ of the retaining piles affected by the reverse fault. A larger *η* value indicates a greater influence of the reverse fault on the maximum deformation of the retaining piles near the pit. Based on the numerical model experiments with 64 different combinations of fault parameters, the data fitting results showed a good linear relationship between *U*_*hm*_ and *η*. Therefore, the predictive model for the maximum deformation of the retaining piles in the influence zone of the footwall affected by the reverse fault is given by:

8 $$U_{hm} = J_{1} \frac{\pi \theta T}{{180^{^\circ } S}} + J_{2}$$

**Table 5:** Factor level table.

Factors levels	*T *(mm)	*θ *(°)	*S* (m)
1	3	45	15
2	9	62	20
3	18	72	25
4	27	82	30

*J*_*1*_ and *J*_*2*_ are relevant variables that can be obtained by fitting experimental data. *θ* is the fault dip angle in degrees, *T* is the fault slip in millimeters, and *S* is the fault position in meters.

**Figure 20:**
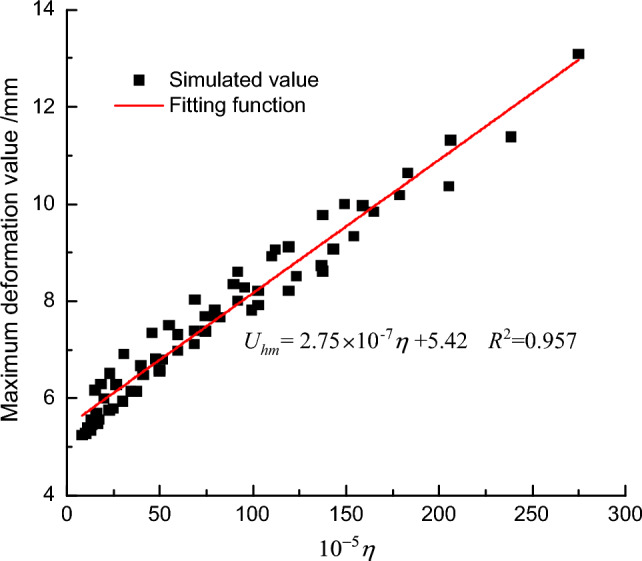
Prediction curve of maximum deformation of retaining piles under the influence of a reverse fault.

As shown in Fig. 20, the predictive equation for the maximum deformation of the retaining piles in the influence zone of the footwall affected by the reverse fault in this project is obtained as follows:

9 $$U_{hm} = 2.75 \times 10^{ - 7} \frac{\pi \theta T}{{180^{^\circ } S}} + 5.42$$

The predictive model for the maximum deformation of the retaining piles, obtained in this section, serves as a reference for predicting the maximum deformation of retaining piles under similar geological conditions caused by various combinations of fault parameters.

## Conclusion and outlook

This paper, based on a deep excavation project in Shenzhen, utilizes the MIDAS GTS NX finite element analysis software to investigate the influence of reverse faulting on the deformation of foundation pit retaining piles. The following conclusions have been drawn:

1. The compressive action of the reverse fault can lead to increased deformation of the retaining piles due to unloading during excavation. The trend of increased deformation shifts towards the upper part of the pile, with the upper part of the pile experiencing more significant deformation compared to the lower part. The overall deformation of the pile follows an approximately spoon-shaped curve distribution, with the maximum deformation occurring in the upper part of the excavation pit.At this point, the maximum horizontal displacement is 7.15 mm.
2. The comparative analysis between simulated values and on-site measured values indicates a good alignment between the numerical trends of the two, thereby validating the reliability and feasibility of the finite element calculations.
3. The deformation of retaining piles under the influence of a reverse fault is directly proportional to the fault slip amount and fault dip angle, and inversely proportional to the distance between the fault and the foundation pit. The variation rate of the maximum deformation of the pile body, *r* (Δ*Z*_*max*_/Δ), increases approximately exponentially with an increase in the fault slip amount and dip angle, and decreases approximately logarithmically with an increase in the distance from the fault to the pit.
4. Sensitivity analysis of the maximum pile deformation to three parameters—fault slip amount, fault dip angle, and fault position—revealed that the fault dip angle had the highest sensitivity, followed by the fault slip amount, while the influence of the fault position was the smallest.
5. Through fitting the data from 64 sets of orthogonal experiments, it was found that the maximum pile deformation *U*_*hm*_ had a good linear relationship with the indicator *η*($\theta \pi T/180^{^\circ } S$), thereby establishing a predictive model for the maximum deformation of foundation pit retaining piles in the influence zone of the footwall affected by a reverse fault. This model provides valuable reference for predicting the maximum pile deformation under similar geological conditions with different combinations of fault parameters.

These research findings can serve as a valuable reference for the study of foundation pit engineering under similar geological conditions. Future research can be expanded to include various foundation pit projects under different geological conditions, thereby broadening the scope of the study and validating the applicability of the research results. Additionally, further exploration of the influence of different numbers and positions of faults on pile deformations can enhance our understanding of the complex interactions between faults and retaining structures.

## Data Availability

The datasets used and/or analyzed during the current study are available from the corresponding author on reasonable request. The materials used in this study are commercially available or can be obtained from the corresponding author upon request. In addition, all relevant codes and protocols used in the study are available on GitHub and can be freely accessed and used for non-commercial purposes. The data that support the findings of this study are available from Shenzhen Dasheng Surveying Technology Co. but restrictions apply to the availability of these data, which were used under license for the current study, and so are not publicly available. Data are however available from the authors upon reasonable request and with permission of Shenzhen Dasheng Surveying Technology Co.
